# Design of a nasopharyngeal tamponade device in canine cadavers

**DOI:** 10.1111/vec.13427

**Published:** 2024-11-21

**Authors:** Anais Allen‐Deal, Joanna Lodzinska, Ingrid Isaac, Efa Llewellyn, Adam Gow, Craig Breheny

**Affiliations:** ^1^ Hospital for Small Animals, Royal (Dick) School of Veterinary Studies University of Edinburgh Edinburgh UK

**Keywords:** canine, epistaxis, hemorrhage, nasopharynx

## Abstract

**Objective:**

To evaluate the feasibility of an improvised tamponade device allowing direct pressure to be applied to the canine nasopharynx.

**Design:**

Proof‐of‐concept study using 8 canine cadavers.

**Methods and Results:**

A tamponade device was made by placing a condom over a nasogastric tube and suturing it to match the length of the nasopharynx. The device was placed in the nasopharynx of canine cadavers via the nares and filled with diluted ioversol. Placement was then confirmed with radiography or computed tomography. Concentrated ioversol was infused into the rostral nasal cavity to assess for a nasopharyngeal seal, defined as no ioversol passing the device seen on imaging. Subjective assessment of adequate nasopharyngeal compression via digital palpation of the soft palate agreed with imaging findings. Repositioning was required in several cases, but with digital palpation, initial placement was more accurate. Subsequent effective placement and a nasopharyngeal seal were achieved in all 8 cadavers.

**Conclusion:**

This device can be placed in the canine nasopharynx, and an adequate nasopharyngeal seal can be achieved, allowing direct compression of the nasopharynx and filling of the nasal cavity with solution. Confirmation of placement was successful with digital palpation and imaging. Further studies are required to investigate the use of this device in live patients.

**Abbreviations**
CT: computed tomography
NG: nasogastric

## 1 INTRODUCTION

Epistaxis can result from disease localized to the nasopharynx, nasal cavity, or nostril (eg, neoplasia, fungal infection, foreign bodies, and trauma) or from systemic diseases (eg, hypertension, thrombocytopenia). Epistaxis was responsible for 0.3% of canine emergency visits at 1 center, with primary nasal disease comprising 78%–83% of cases.^1^, ^2^ Additionally, epistaxis is a potential complication after rhinoscopy‐assisted biopsy, with 1 study reporting that 1.8% of dogs have an undefined protracted hemorrhage.^3^ Hemorrhage is best managed by applying direct pressure to the site of bleeding, which can be difficult to achieve.^4^

Applying direct pressure to the site of hemorrhage is challenging in cases of epistaxis because neither the nasal cavity nor the nasopharynx is accessible, which allows for traditional methods of compression. A variety of techniques have been described to stop epistaxis in veterinary medicine, including packing the nasal cavity with sponges, applying cold compresses, ligation of the ipsilateral carotid artery, and injection of an embolic agent into feeding arteries.^5^ Epinephrine or tranexamic acid may be instilled into the nasal cavity via the nares as an alternative topical treatment. Anecdotal evidence suggests that these are widely used; however, there are no data regarding their efficacy in veterinary medicine. The difficulty with these medications being able to reach their target site, along with the limited volume that can be administered via the nasal cavity, brings into question the efficacy of these treatments.

Clinical practice guidelines for human medicine recommend nasal packing in epistaxis that has not stopped after 5 minutes of external compression of the nose.^6^ A variety of different nasal packing devices exist to allow pressure to be applied to the affected area to achieve hemostasis. Devices such as the Rapid Rhino can be inserted into the human nasal cavity and inflated to apply direct pressure.^7^ No commercial veterinary devices have been specifically developed for management of epistaxis. The nasal anatomy in different dog breeds varies and poses a challenge to create a “one size fits all” device. Despite not being considered a common site of bleeding, the nasopharynx may provide a more reliable target for compression. Veterinary literature is currently limited to 1 case report detailing the placement of an inflated balloon catheter within the nasopharynx and rostral nasal cavity to allow for stabilization until the right common carotid artery could be ligated.^8^

The objective of the current study was to evaluate the feasibility of a makeshift device designed to be placed into the canine nasopharynx, and inflated in situ, to allow direct pressure to be applied to the nasopharynx, creating a tamponade. Such a device could have 2 functions: compression of bleeding vessels in the nasopharynx while the seal within the nasopharynx allows solutions, such as hemostatic medications, to be instilled into the nasal cavity without leaking caudally, therefore increasing contact time.

## 2 MATERIALS AND METHODS

### 2.1 Cadavers

This prospective study used canine cadavers donated with owner consent for teaching and research purposes. Approval was granted by the Veterinary Ethics in Research Committee of the authors’ university. Canine cadavers were considered for study inclusion if they had been refrigerated for less than 7 days and had never been frozen. Canine cadavers were excluded if they had preexisting nasal disease or head trauma. The breed, age, and body weight of each cadaver were recorded. Ten canine cadavers were used in this study. Two cadavers were used as pilot studies. The first pilot cadaver was used to assess whether the device could be successfully placed and identified on radiography. The second was used to assess which measurements were required for placement and how best to confirm tamponade. The remaining 8 cadavers were used to refine the technique.

### 2.2 Measurements

Measurements to guide placement of the device were made on radiography or computed tomography (CT). The initial cadavers had a head CT performed in sternal recumbency using a helical, 64‐slice CT^a^. The final 3 cadavers had lateral radiographs taken of the head and neck. The distance from the planum nasale to the level of the presphenoid bone just rostral to the pterygoid process and approximately at the midsection of the soft palate and the length of the nasopharynx from the caudal margin of the hard palate to the level of the presphenoid bone just rostral to the pterygoid process were measured on the sagittal plane of the CT or on the lateral radiograph.

### 2.3 Device preparation

**FIGURE 1:**
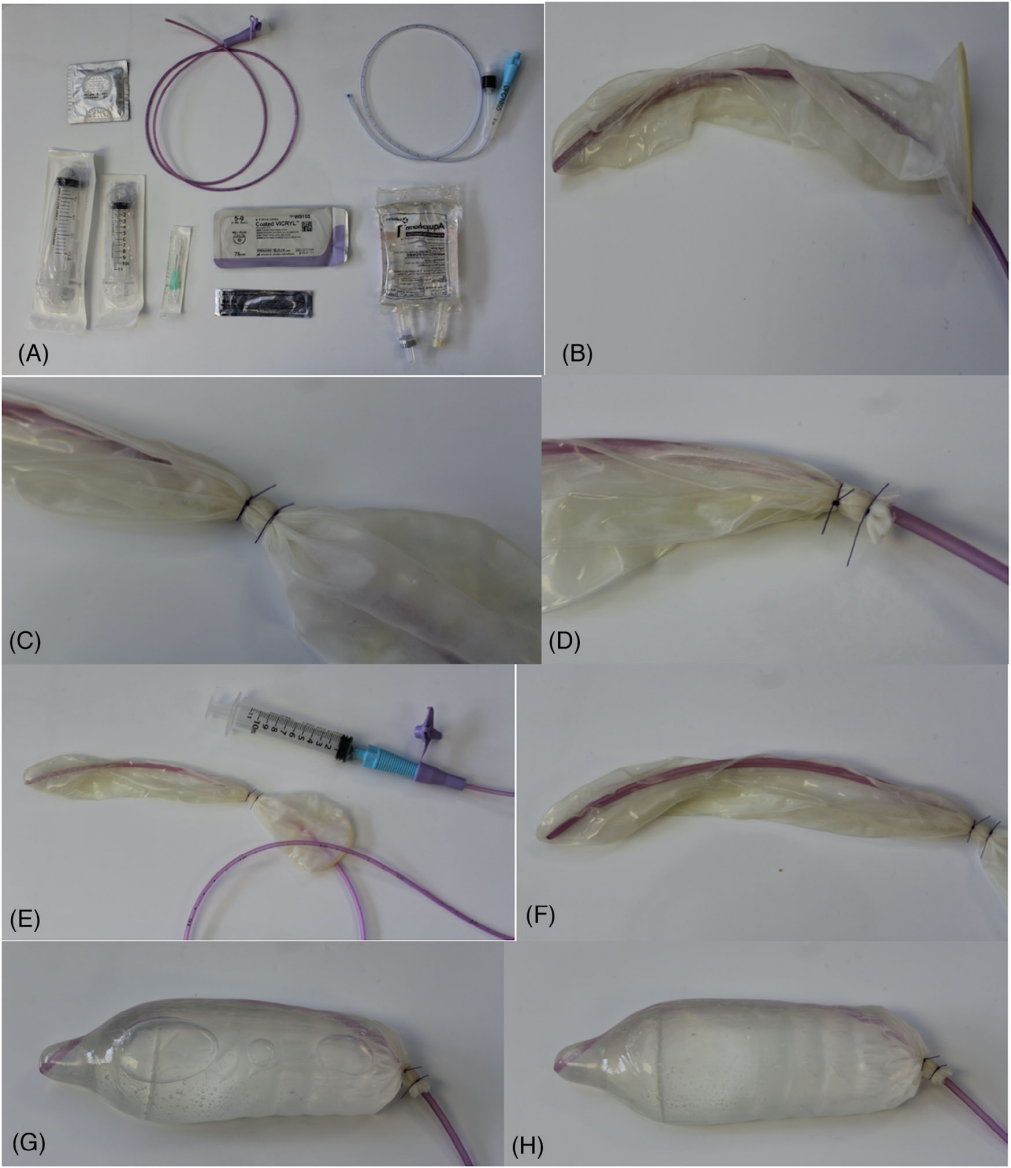
(A) Equipment required to place the tamponade device. (B) A condom is placed over the nasogastric tube. (C) Two circumferential sutures are applied at the premeasured point. (D) The residual condom distal to the sutures is removed with a scalpel. (E) The guidewire is removed, and an adaptor is attached to the tube. (F) Negative pressure is applied to remove any residual air within the tamponade device. (G) Saline is instilled to fill the condom, ensuring that there is no leakage. (H) The condom is held vertically with the tip uppermost, and air within the condom is removed.

A condom^b^ was placed over the tip of a 6‐Fr nasogastric (NG) tube^c^. Using a 2‐0 polyglactin 910 multi‐braided suture^d^, 2 encircling single‐interrupted sutures were tightly placed around the condom, creating a watertight seal at the length to match the measured length of the nasopharynx. Excess condom material was removed with a number 15 scalpel blade to facilitate placement. The guidewire from the NG tube was removed, and an adaptor was attached to allow instillation of liquids. Air remaining in the condom was removed to prevent creation of a gas lucency on radiography or CT, which makes confirmation of placement and tamponade more challenging. Water was instilled into the NG tube to fill the condom and ensure that a watertight seal could be achieved, acting as a leak test. The water was then removed with the tube and tip held vertically to remove any residual air (Figure 1).

### 2.4 Placement

In the current study, both CT and radiography were used for measurements and for placement of the device. We determined that both imaging modalities were equally effective; therefore, placement here is described in a cadaver where radiography and digital palpation were used to confirm device placement. This method is likely to be translatable to all types of veterinary practice.

**FIGURE 2:**
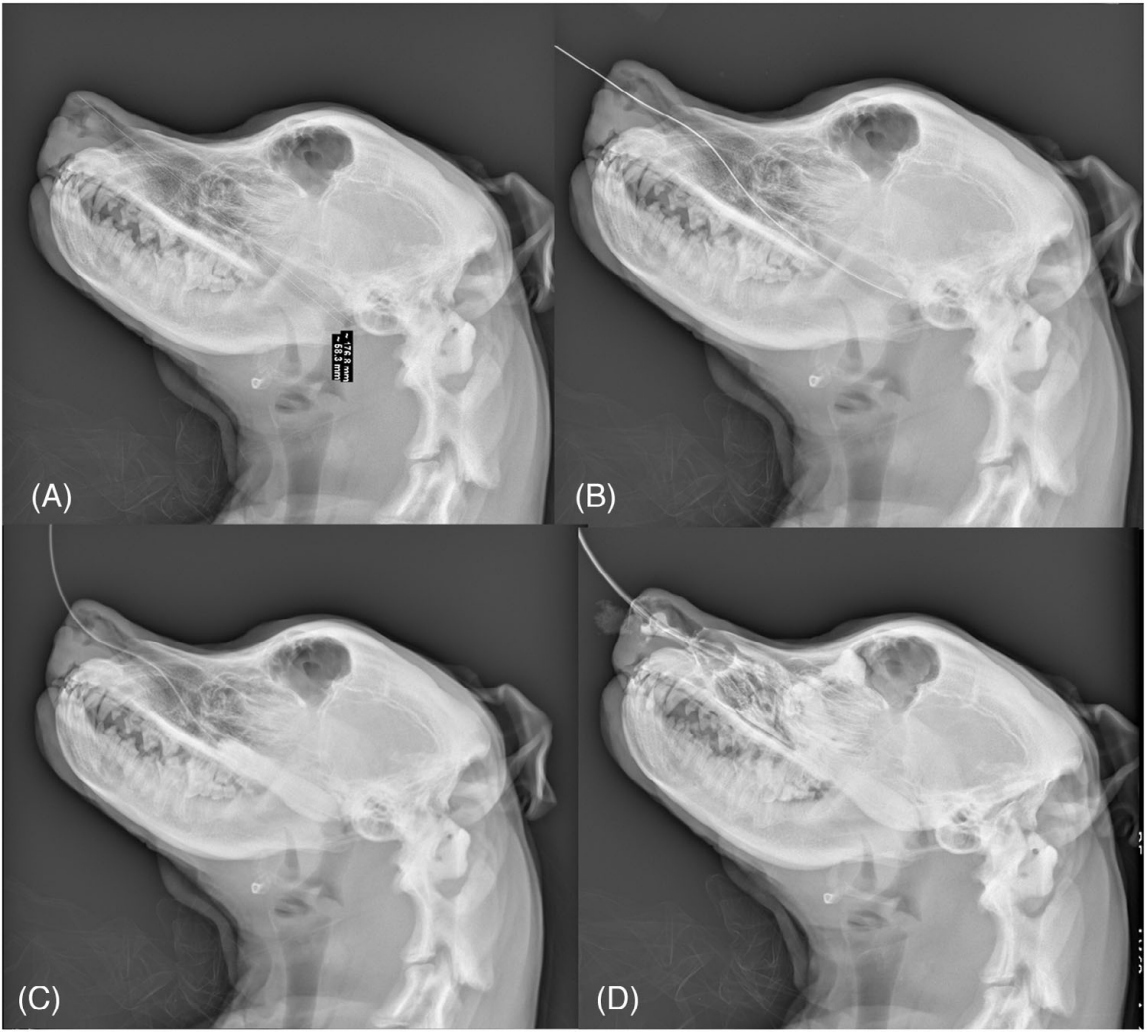
Lateral head radiographs show (A) measurement of the nasopharynx; (B) the nasogastric tube in situ; and (C) the condom filled with 20% solution of the contrast medium in the correct position. (D) A repeat radiograph after the instillation of 50% diluted ioversol solution via an inflated Foley catheter. The nose is tilted upward to determine if the contrast would migrate caudally to the device. No contrast medium leakage caudal to the device was observed.

The cadaver was placed in left lateral recumbency, and a radiograph of the head and neck was taken (Figure 2). The distance from the planum nasale to the caudal margin of the nasopharynx and the length of the nasopharynx were measured. The device was passed through the nostril of the cadaver through the ventral meatus, by aiming ventrally and medially, to the caudal end of the nasopharynx based on the length measured from the radiograph. A second left lateral head and neck radiograph was taken to confirm that the device was in the caudal nasopharynx (Figure 2). Successful placement was considered when the device was passed through the nares and ended at the caudal border of the nasopharynx, without migration into the oropharynx or displacement into other parts of the nasal anatomy.

A diluted contrast solution^e^ was made with 1 part ioversol^e^ (350 mg iodine/mL) to 4 parts 0.9% saline, referred to as 20% diluted ioversol in this study. This was instilled into the condom via the NG tube, initially using 0.5 mL/kg body weight of the cadaver because a previous study estimated the canine nasopharynx volume to be 0.48 cm^3^/kg.^9^ The firmness of the soft palate was subjectively assessed during instillation by palpating the oral cavity, which determined the volume required. The oral cavity was held open, and the caudal pharynx was visually inspected for caudal displacement of the device. A third left lateral head and neck radiograph was taken to assess if the device was in an appropriate position and the volume instilled was sufficient (Figure 2). The device placement and the degree of fill were then evaluated by digitally applying pressure to the soft tissues of the nasopharynx.

A 6‐Fr Foley catheter^f^ was inserted into the nasal cavity blindly via the contralateral nostril and inflated. Cranial traction was then applied to the Foley catheter until it ceased movement, which resulted in the Foley catheter balloon sitting in the most rostral portion of the nasal cavity. The cadaver's nose was then tilted up until it was approximately 30 degrees in relation to the neck, and 50% diluted ioversol (1 part ioversol [350 mg/mL], 1 part 0.9% saline) was instilled rostral to the tamponade device via the Foley catheter. This dilution of ioversol could be distinguished from the 20% diluted ioversol within the condom on imaging, making it possible to determine if 50% diluted ioversol had passed caudal to the inflated condom. A fourth radiograph was taken (Figure 2). No contrast had passed the condom, confirming a successful nasopharyngeal seal that allowed the nasal cavity to be filled with a solution and provided direct contact with the nasal cavity and turbinates.

### 2.5 Statistics

Descriptive statistics were performed in this study. Summary statistics for categorical variables (body weight, length of the nasopharynx, length of NG tube required to reach the caudal border of the nasopharynx, and volumes of ioversol) are presented as median and range.

## 3 RESULTS

All cadavers used were older than 6 months of age and included 4 crossbreeds, 3 Labrador Retrievers, 1 Cavalier King Charles Spaniel, 1 Border Collie, and 1 Greyhound. The median body weight was 24.5 kg (range: 8.0–32.9 kg).

Eight cadavers had placement of the device; 5 of those had a successful initial placement. CT was used for placement in 5 cadavers, and radiography was used in 3.

The median length of the nasopharynx was 5.6 cm (range: 4.5–6 cm). The median length of NG tube required to reach the caudal border of the nasopharynx was 17.5 cm (range: 12–23 cm).

Three cadavers required repositioning of the device. In 1 cadaver, the device was reported to be displaced into the nasal turbinates. In the next cadaver, displacement of the device occurred, but the location of the displacement was not recorded. In the third cadaver, the condom migrated caudally when inflated. The last cadaver required the placement of Doyen forceps on the external part of the NG tube immediately rostral to the nares to prevent further caudal migration. These 3 cadavers then had subsequent successful placement of the device. All 8 cadavers achieved an effective seal of the nasopharynx. The median volume required to create an effective seal in these 8 cadavers was 0.51 mL/kg (range: 0.38–1.24 mL/kg).

## 4 DISCUSSION

This study confirmed the feasibility of placing a low‐resource device in the nasopharynx of canine cadavers, which could be used for the management of epistaxis. Readily available materials were purposely used to ensure this was feasible in a wide variety of veterinary practices. In the authors’ institute, the price of placing this device, including the required radiographs and general anesthesia, would be approximately two thirds the cost of transfusing 1 unit of packed red blood cells.

The current study evaluated 2 of the device's uses. It can be successfully placed into the nasopharynx and inflated, creating a seal or tamponade of the nasopharynx that would allow compression of any bleeding vessels in the unlikely event of a nasopharyngeal bleed. More vitally, the seal created by this device caudally allows solutions to be instilled into the nasal cavity more effectively than current methods. These solutions would have an increased chance of making contact with the site of bleeding with a more prolonged dwell time, theoretically improving their performance. Vasoactive or hemostatic agents, such as epinephrine or tranexamic acid, could be instilled in live patients for a more consistent and prolonged contact with the source of hemorrhage, thus maximizing the chances of hemostasis.

Studies in people have reported variability in the volumes required to inflate devices such as the Rapid Rhino, which sit in the nasal cavity. To overcome this complication, Mackeith et al^10^ suggested using a manometer to confirm filling of the Rapid Rhino. In the current study, the device was placed in the nasopharynx, but the volumes required to fill the device still varied. Although a nasopharyngeal seal was demonstrated in this study, the pressure exerted by the device may not be sufficient to compress vasculature and cease hemorrhage (ie, tamponade). It may also be that the exerted pressure decreased with time, but the current study did not assess this. The use of manometers would have allowed measurement of the pressure created within the device and should be evaluated in future studies. Further work would be required to determine whether manometers would be appropriate for assessing tamponade for this device. In the interim period, manual palpation through the oral cavity and the use of contrast imaging modalities can be used to confirm adequate placement.

Limitations of the described technique include that it has been developed through the use of cadavers, which were not actively hemorrhaging nor did they have intranasal disease, which would be the patient populations to which this is of the most interest. Placement may be less successful in live patients with intranasal disease or ongoing hemorrhage, limiting the device's use. Only 1 cadaver had brachycephalic conformation, and further work is required to see if placement could be consistently achieved in brachycephalic patients. Prospective studies are recommended to see if this device is effective at ceasing hemorrhage in canine patients experiencing epistaxis.

Additional limitations of this device include that it may cause discomfort and prevent the patient's ability to breathe through their nose. A mouth gag would be required to prevent the operator from being bitten during digital palpation. There is a risk of oropharyngeal obstruction and a lack of a standardized volume to fill the nasopharynx. For these reasons, we would suggest that this device be restricted to anesthetized and endotracheally intubated patients. Although this may mean it is not suitable for all patients, the device could have clinical use in a subset of patients suffering from uncontrolled epistaxis, including those hemorrhaging from the nasal cavity or nasopharynx after biopsy, where classic methods of hemostasis are limited.

In conclusion, this study shows that a low‐resource tamponade device could provide an inexpensive and widely available method of achieving hemostasis in a population where hemorrhage is difficult to manage. Further studies would be required to evaluate if this procedure can be repeated in live dogs experiencing epistaxis. This device would be an optimal step in achieving hemostasis without having to escalate to invasive procedures such as the injection of embolic agents or ligation of the carotid artery.^5^

## AUTHOR CONTRIBUTIONS

**Anais Allen‐Deal**: Data curation; investigation; methodology; writing—original draft; writing—review and editing. **Joanna Lodzinska**: Investigation; methodology; resources; software; writing—review and editing. **Ingrid Isaac**: Investigation. **Efa Llewellyn**: Conceptualization; writing—review and editing. **Adam G Gow**: Conceptualization; writing—review and editing. **Craig Breheny**: Conceptualization; methodology; project administration; supervision; writing—original draft; writing—review and editing.

## CONFLICT OF INTEREST STATEMENT

The authors declare no conflicts of interest.

## ENDNOTES

a Somatom Definition AS Siemens, Erlangen, Germany.

b Durex Extra Safe, Durex, Slough, UK.

c Weighted Nasogastric Plus Feeding Tubes with Flushing stylet, Mila International Inc., Florence, KY.

d Vicryl, Ethicon Inc, Johnson & Johnson, Raritan, NJ.

e Optiray, Guerbet, Villepinte, France.

f Foley Catheter without Wire Stylet, Mila International Inc., Florence, KY.
